# Vesicle transporter GOLT1B mediates the cell membrane localization of DVL2 and PD-L2 and promotes colorectal cancer metastasis

**DOI:** 10.1186/s12935-021-01991-z

**Published:** 2021-05-31

**Authors:** Tengfei Liu, Binbin Liu, Yiting Liu, Xingzhi Feng, Xuefei Jiang, Jiahui Long, Qianling Gao, Zihuan Yang

**Affiliations:** Guangdong Provincial Key Laboratory of Colorectal and Pelvic Floor Diseases, Guangdong Institute of Gastroenterology, The Sixth Affiliated Hospital of Sun Yat-Sen University, Guangzhou, 510655 Guangdong China

**Keywords:** GOLT1B, CRC, Metastasis, PDX, DVL2, PD-L2

## Abstract

**Background:**

Colorectal cancer (CRC) is the third most diagnosed and second leading cause of cancer death worldwide. Hallmark proteins processing is usually dysregulated in cancers. Finding key regulatory molecules is of great importance for CRC metastasis intervention. GOLT1B is a vesicle transport protein which is involved in cytosolic proteins trafficking. However, its role in cancer has never been addressed.

**Methods:**

CRC cell lines and subcutaneous xenograft animal model were utilized to investigate the biological function of GOLT1B. Patients samples were used to validate the correlation between GOLT1B and clinical outcome. In vivo targeted delivery of GOLT1B-siRNA was investigated in PDX (Patient derived tumor xenograft) model.

**Results:**

We found that GOLT1B was highly expressed in CRC, and was an independent prognostic marker of overall survival (OS) and progression free survival (PFS). GOLT1B could promote CRC metastasis in vitro and in vivo. GOLT1B overexpression could increase DVL2 level and enhance its plasma membrane translocation, which subsequently activated downstream Wnt/β-catenin pathway and increase the nuclear β-catenin level, hence induce epithelial-mesenchymal transition (EMT). In addition, GOLT1B could also interact with PD-L2 and increase its membrane level. Co-culture of GOLT1B-overexpresed CRC cells with Jurkat cells significantly induced T cells apoptosis, which might further promote cancer cell the migration and invasion. Further, targeted delivery of GOLT1B siRNA could significantly inhibit tumor progression in GOLT1B highly expressed PDX model.

**Conclusion:**

Taken together, our findings suggest that the vesicle transporter GOLT1B could promote CRC metastasis not only by assisting DVL2 translocation and activating Wnt/β-catenin pathway, but also facilitating PD-L2 membrane localization to induce immune suppression. Targeted inhibition of GOLT1B could be a potential therapeutic strategy for CRC treatment.

**Supplementary Information:**

The online version contains supplementary material available at 10.1186/s12935-021-01991-z.

## Introduction

Colorectal cancer is now the third most common malignant tumor worldwide, and distant metastasis is the main cause of its death [1, 2]. Clarifying the key molecules related to CRC metastasis and prognosis and their regulatory mechanisms is the key to the treatment of CRC.

Protein localization is very important for the maintenance of their normal function. For example, DVL2 are recruited to the membrane receptor complex to initiate downstream Wnt signal cascade [3, 4]. Programmed death-ligand 2 (PD-L2) expressed on the surface of cancer cells could induce immunosuppression through interaction with PD-1 in immune cells [5, 6], and blocking this interaction gave rise to an impressive clinical benefit in various of cancers [7–14]. Aberrant localization of hallmark proteins can alter their function so that their ability to suppress cancer or induce cancer metastasis is changed. Therefore, the mislocalization of such proteins could serve as novel therapeutic targets of cancer.

Golgi apparatus is a crucial cell component responsible for transporting, modifying, and packaging proteins into vesicles for delivery to targeted locations. Studies have shown that Golgi-related genes play important roles in regulating cancer occurrence and development. Golgi phosphorylated protein 2 (GOLPH2) dysregulation has been reported in many cancers, including oral squamous cell carcinoma, hepatocellular carcinomas, and esophageal cancer [15–17]. Golgi phosphorylated protein 3 (GOLPH3) promotes the development of CRC and induce resistance to 5-FU by mediating the mTOR pathway [18–20]. Golgi membrane protein 1 (GOLM1) expression is correlated with early recurrence, metastasis, and poor survival of HCC patients [21, 22]. Golgi vesicle transporter 1A (GOLT1A) has been shown to regulate tamoxifen sensitivity in breast cancer and promote cell proliferation in lung cancer [23, 24]. However, the function of GOLTIB, which belongs to the same family, has never been addressed. Little is known about the mechanism of Golgi-related proteins in regulating cancer metastasis.

In the present study, we found that the vesicular transporter GOLT1B, also known as Golgi transport 1 homolog B, was overexpressed in CRC. High GOLT1B is significantly correlated with poor prognosis. In vitro and in vivo experiments showed that GOLT1B could promote CRC cells migration and invasion. As to the mechanism, GOLT1B could interact with DVL2 and facilitate its transportation to the cell membrane, where it can bind to the cytoplasmic C-terminus of frizzled receptor, leading to the activation of Wnt signaling and epithelial-mesenchymal transition (EMT). In addition, we found that GOLT1B can also interact with PD-L2 and induce T-cell apoptosis. Our results suggest that GOLT1B is very important in regulating protein transportation and localization. GOLT1B may be a predictive marker and therapeutic target for metastatic CRC.

## Materials and methods

### Cell lines and cell culture

Human CRC cell lines (HCT116, RKO) were purchased from the American Type Culture Collection (ATCC). The STR genotyping data of the two cell lines are consistent with the ATCC database. HCT116 was cultured with McCoy's 5a Medium Modified (Gibco, USA) medium containing 10% fetal bovine serum (Gibco, USA), RKO was cultured with MEM (Gibco, USA) medium containing 10% fetal bovine serum (Gibco, USA), Jurkat T lymphocyte cell line were purchased from the China Center for Type Culture Collection (Shanghai, China). Cells were maintained in RPMI1640 medium supplemented with 10% fetal bovine serum and placed in a humidified incubator at 37 °C with 5% CO_2_.

### Primers and antibodies

All the primers used are listed in the supplemental data (Additional file 1: Table S1). T-PER tissue protein extraction reagent (Thermo, Rockford, IL, USA), protease inhibitor group III and phosphatase inhibitor group II (Millipore, Germany) were used for protein preparation. Proteins were quantified using BCA kits (Thermo, USA). Antibodies used are anti-GOLT1B (Affinity Biosciences, DF9071), anti-DVL2 (CST, #3224), anti-β-catenin (CST, #25362), anti-GSK3β (CST, #9832), anti-pGSK3β(ser9) (CST, #5558), anti-TCF1/TCF7 (CST, #2203), anti-TCF4/TCFL2 (CST, #2569), anti-PD-L2 (CST, #82723), anti-flag (Sigma, F1804), anti-PCNA (Proteintech Group, # 10205–2-AP) and anti-GAPDH (Proteintech Group, #10494–1-AP).

### Patient samples

Paired normal tissue (5 cm from the tumor boundary), paracancerous (2 cm from the tumor boundary) and tumor tissues were obtained from 8 CRC patient from the Sixth Affiliated Hospital of Sun Yat-sen University. Tissue microarray (TMA) of primary CRC from 224 patients were constructed. Freshly resected tumor tissue from 3 CRC patients were used to construct PDX model. None of these patients received adjuvant chemotherapy or radiotherapy before surgury. Studies were approved by the Human Medical Ethics Committee of the Sixth Affiliated Hospital of Sun Yat-sen University.

### Plasmid construction and siRNA transfection

The cDNA ORF of human GOLT1B was amplified and cloned into the pcDNA 3.1(+) plasmid by homologous recombination using In-fusion HD cloning kit (Clonetech, Tokyo, Japan). The constructed plasmid was used for transient transfection. The plasmid was transfected into CRC cells using Lipofectamine 3000 (Invitrogen, USA) according to the manufacturer's instructions and the cells were collected 48 h after transfection for experiments. In this study, the full-length human GOLT1B cDNA ORF was constructed by homologous recombination into the lentivirus expression vector pCDH-CMV-MCS-EF1-copGFP (SBI Pharmaceuticals, Tokyo, Japan) for the construction of stable cell lines. HEK 293T cells were co-transfected with pCDH-GOLT1B/pCDH-Vector, pCMV-δ8.91 and pCMV-VSVG using Lipofectamine 3000 to produce lentiviruses. The medium was changed 24 h after transfection and virus supernatants were collected 48 h and 72 h after transfection. After ultracentrifugation, the supernatant was concentrated and added to luciferase-expressing HCT116. The virus-infected HCT116 cells were selected with puromycin. All plasmid constructions were confirmed by sequencing. The expression effect was also verified by qPCR and western blot. The primers used in plasmid construction are listed in Additional file 1: Table S1. The siRNA for GOLT1B was synthesized by RiboBio (Guangzhou, China). CRC cells were transfected with siRNA using Lipofectamine™ RNAiMAX (Invitrogen, USA). After 48 h of transfection, the cells were used for experiments such as western blot, migration and invasion.

### RNA-seq analysis

Total RNA from CRC cells transfected with scramble RNA or si-GOLT1B were extracted by Trizol method, respectively. RNA-seq was completed by Beijing Genomics Institute (BGI-tech) on BGISEQ-500 platform [25]. KEGG, GO and GSEA analyses were performed on Dr. Tom platform (BGI-tech, China) to determine the genes and signal pathways related to GOLT1B knockdown in CRC cells.

### Immunofluorescence

Cells cultured on coverslips were washed in PBS for 3 times, and then fixed with 4% paraformaldehyde at room temperature for 30 min. Then the fixed cells were permeabilized in 0.5% Triton X-100 for 10 min, and blocked with 5% bovine serum albumin at room temperature for 2 h. Cells were incubated with primary antibody at 4 ℃ overnight. On the second day, fluorescent secondary antibody was added, and the nucleus was counterstained with DAPI. Images were analyzed using SP8 (Leica, Germany) confocal microscope.

### Cell migration and invasion assays

For wound healing assay, the Ibidi culture insertion chambers (Ibidi, Germany) were placed into 12-well plates (Thermo Fisher Scientific) and 1.0 × 10^5^ CRC cells were added into each well. When the cells grow to full confluence, the chambers were taken out. The floating cells were washed out with PBS and FBS-free culture medium were then added. Culture plates were placed in the Incuyte Zoom System (Essen Bioscience, USA) for photographing to observe the wound healing process. The area covered by the wound was measured using ImageJ software.

Cell migration and invasion were determined by transwell assay. Breifly, CRC cells resuspended in serum-free medium were added to the upper well of transwell chambers and cultured for 48 h. For invasion assay, Matrigel (Corning, USA) was diluted at 1:10 with serum-free culture medium and coated to the upper surface of the transwell chambers before cell seeding. The cells were fixed with 4% paraformaldehyde for 30 min and stained with 0.1% crystal violet. Five fields of view were randomly photographed under a 200-fold microscope. The number of cells was then analyzed using Image J.

### TMA Immunohistochemistry (IHC)

TMA sections were dewaxed and rehydrated. Antigen retrieval was achieved by Tris–EDTA (pH 9.0) boiling for 10 min. Immunostaining of the TMA sections were assessed independently by two pathologists. To evaluate the GOLT1B expression level, each section was assigned a score based on intensity and positive area. The intensity was scored from 0 to 3 (0 with no staining, 1 with weak staining, 2 with moderate staining, 3 with strong staining). The scores based on the percentage of positive cancer cells is defined as follows: 1 (0–25% of positive cells), 2 (26–50% of positive cells), 3 (51–75% of positive cells), 4 (76–100% of positive cells). We multiplied the two scores to obtain a final score (ranging from 0 to 12). Then we ranked the tissue samples by the final score. Finally, X-tile was used to determine the optimal cutoff point for dividing samples into two groups: samples with scores ≥ 5 defined as GOLT1B high expression, scores < 5 defined as GOLT1B low expression.

### Luciferase reporter assay

The Wnt pathway reporter plasmid pGL4․49 [luc2P⁄TCF-LEF RE⁄Hygro] was purchased from Promega (Madison, USA). The pRL-TK and the pcDNA3.1 (+)-GOLT1B overexpression plasmid were co-transfected into CRC cells using Lipofectamine 3000. After 24–48 h, luciferase activity was determined using a double luciferase reporter gene analysis system (Promega, E1910, USA) according to the manufacturer's instructions.

### Animal studies

Four-week-old female BALB/C nude mice were purchased from VitalRiver Laboratory Animal Technology Co., Ltd. (Beijing, China) and reared under SPF-grade sterile conditions in the Laboratory Animal Center of the Sixth Affiliated Hospital of Sun Yat-sen University.

For liver metastasis model, 5.0 × 10^5^ luciferase-labeled HCT116 cells (HCT116^Vector^ and HCT116^GOLT1B^) were injected into the spleen. Bioluminescence signals were monitored weekly using an In vivo imaging system (IVIS, Perkinelmer, Germany). Mice were sacrificed and the number of liver metastases were counted 7 weeks after inoculation. The liver was fixed with 4% paraformaldehyde and embedded in paraffin for further analysis (IHC, hematoxylin–eosin).

For PDX model, freshly resected colorectal cancer tumor tissue was inoculated into the right anterior axilla of BALB/c nude mice with abundant blood supply to construct a PDX model (3 cases, 8 mice in each case). The pathological information of colorectal cancer patients used for PDX modeling is in Additional file 2: Table S2. At the same time, we synthesized in vivo targeted siRNA according to siGOLT1B-2 (RiboBio, China). Two weeks after inoculation, mice were divided into two groups. The control group and treatment group were intraperitoneally injected with si-control and siGOLT1B (1 nmol) every 2 days for 4 weeks, respectively. Body weight and tumor size were observed and recorded. When the tumor reached about 1500 mm^3^, animals were sacrificed.

### Statistical analysis

All statistical analysis was performed with SPSS 24.0 (Chicago, IL, USA) or GraphPad Prism 8.0 (GraphPad, La Jolla, CA, USA). The significance of difference between groups was analyzed by two-tailed paired or unparied Student’s t-test, one-way ANOVA or two-way ANOVA, as appropriate. A P-value < 0.05 was considered statistically significant (*P < 0.05, **P < 0.01, ***P < 0.001, ****P < 0.0001).

Patient survival was analyzed by Kaplan–Meier analysis with log-rank test. Univariate and multivariate survival analysis were based on the Cox regression analyses model.

## Results

### GOLT1B is highly expressed in CRC and correlated with poor prognosis

**Fig. 1:**
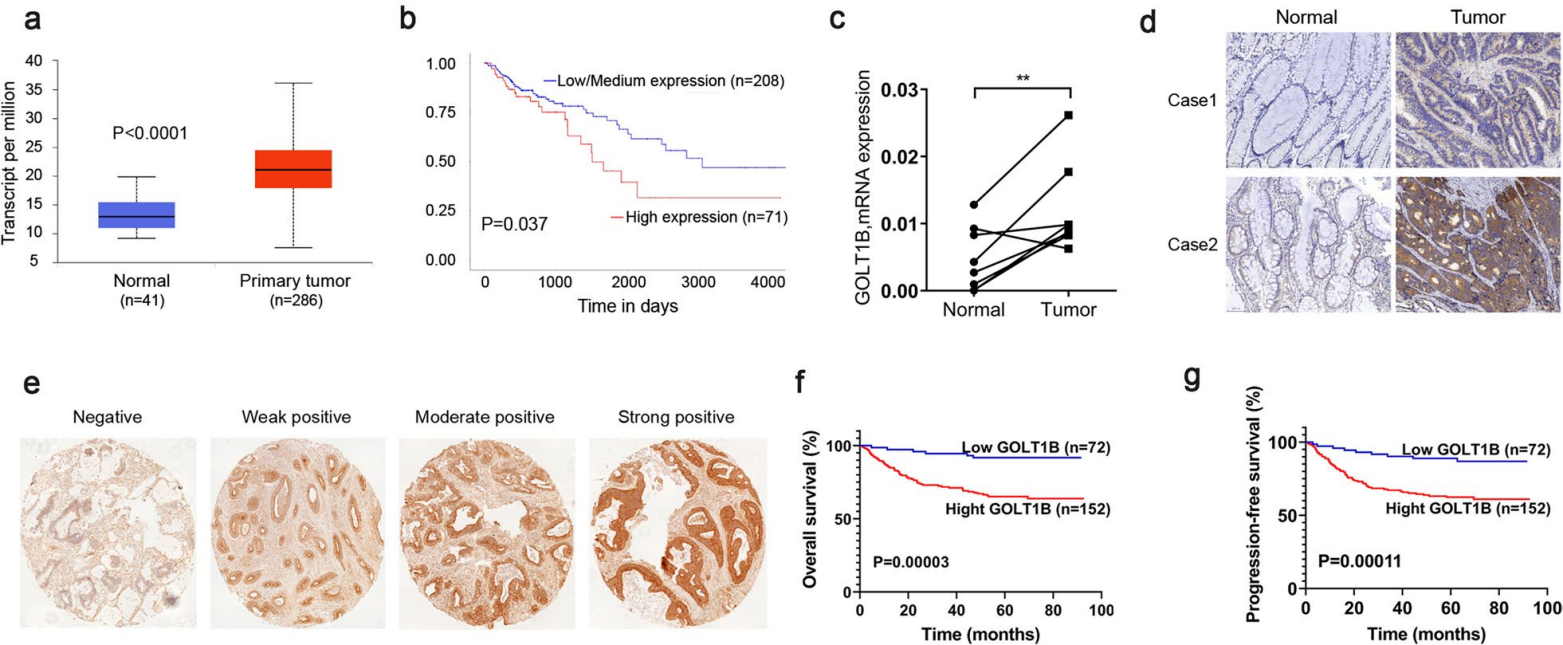
GOLT1B is highly expressed in CRC and predicts poor clinical prognosis. **a** Comparison of GOLT1B expression level in colon adenocarcinoma and normal tissue in UALCAN database. **b** Kaplan–Meier survival curves of OS in CRC patients from UALCAN database. **c** GOLT1B level determined by qRT-PCR (n = 8) and **d** IHC staining in paired CRC tumor tissues and normal tissues. **e.** Representative IHC staining of GOLT1B in a tissue microarray of human CRC tissues. **f**, **g** Kaplan–Meier survival curves of OS and PFS in 224 CRC patients by GOLT1B expression (*P* = 0.00003 and *P* = 0.00011, respectively). Low GOLT1B is defined as IHC score < 5 and High GOLT1B is defined as IHC score ≥ 5

Using TCGA CRC datasets from UALCAN and Oncomine, we found that GOLT1B was highly expressed in CRC and correlated with poor survival (Fig. 1a, b, Additional file 3: Fig. S1a, b) [26–28]. Consistently, GOLT1B level were significantly higher in tumor tissues compared with that in paired normal tissue (P < 0.01) (Fig. 1c, d). IHC examination on tumor tissue microarray of 224 CRC patients showed that GOLT1B is positively stained in the tumor area of most patients. In consist with the TCGA survival analysis, Kaplan–Meier analysis showed that high GOLT1B expression was significant associate with poor overall survival (OS) (P = 0.00003) as well as progression-free survival (PFS) (P = 0.00011) (Fig. 1e–g). Higher GOLT1B IHC score was positively correlated with higher TNM stage and metastasis (Table 1). Univariate and multivariate Cox regression analysis showed that GOLT1B was an independent prognostic marker of OS and PFS, and it was an important indicator of progression and metastasis (Tables 2, 3).

**Table 1:** Correlation between GOLT1B expression and clinicopathological features in CRC patients

Variables	Low GOLT1B (n = 72)	High GOLT1B (n = 152)	*P* value
Gender			1.0000
Male	41 (56.9%)	87 (57.2%)	
Female	31 (43.1%)	65 (42.8%)	
Median age			0.0099**
< 67 years	45 (62.5%)	66 (42.4%)	
≥ 67 years	27 (37.5%)	86 (56.6%)	
pT stage			0.6430
T1	4 (5.6%)	13 (8.5%)	
T2	19 (26.4%)	31 (20.4%)	
T3	41 (56.9%)	91 (59.9%)	
T4	8 (11.1%)	17 (11.2%)	
pN stage			< 0.0002***
N0	63 (87.5%)	98 (64.5%)	
N1	7 (9.7%)	44 (28.9%)	
N2	2 (2.8%)	10 (6.6%)	
pM stage			0.0005***
M0	71 (98.6%)	127 (83.6%)	
M1	1 (1.4%)	25 (16.4%)	
Histological grade			0.3711
G1	3 (4.2%)	8 (5.3%)	
G2	40 (55.6%)	93 (61.2%)	
G3	29 (40.2%)	51 (33.5%)	

All data are shown as numbers and percentages. Low GOLT1B is defined as IHC score < 5 and High GOLT1B is defined as IHC score ≥ 5. *p < 0.05, **p < 0.01, ***p < 0.001, ****p < 0.0001

**Table 2:** Univariate and multivariate analysis of different parameters for overall survival in CRC patients

Variables	Univariate analysis		Multivariate analysis	
	HR (95%CI)	*P* value	HR (95%CI)	*P* value
Gender (male vs female)	0.881 (0.526–1.477)	0.632		
Age (≥ 67 vs < 67)	1.929 (1.135–3.281)	0.015*	1.318 (0.749–2.318)	0.338
pT status (T3-T4 vs T1-T2)	7.270 (2.635–20.057)	< 0.001***	3.004 (1.004–8.989)	0.049*
pN status (N1-N2 vs N0)	7.164 (4.201–12.217)	< 0.0001****	2.690 (1.429–5.065)	0.0022**
pM status (M1 vs M0)	15.165 (8.692–26.456)	< 0.0001****	6.337 (3.387–11.859)	< 0.0001****
Histological grade (G3 vs G1-G2)	0.594 (0.335–1.054)	0.075	1.144 (0.620–2.109)	0.667
Recurrence (YES vs NO)	1.752 (0.701–4.382)	0.230		
GOLT1B expression (High vs Low)	5.089 (2.189–11.834)	< 0.001***	2.520 (1.034–6.142)	0.042*

Univariate and multivariate Cox proportional hazards regression were used to calculate Hazard ratio (HR), 95% confidence intervals (95% CI) and p values in SPSS 24.0. Low GOLT1B is defined as IHC score < 5 and High GOLT1B is defined as IHC score ≥ 5. *p < 0.05, **p < 0.01, ***p < 0.001, ****p < 0.0001

**Table 3:** Univariate and multivariate analysis of different parameters for progression-free survival in CRC patients

Variables	Univariate analysis		Multivariate analysis	
	HR (95%CI)	*P* value	HR (95%CI)	*P* value
Gender (male vs female)	1.075 (0.569–1.519)	0.772		
Age (> 67 vs ≤ 67)	1.452 (0.889–2.372)	0.137		
pT status (T3-T4 vs T1-T2)	3.708 (1.770–7.769)	< 0.001***	1.736 (0.767–3.928)	0.186
pN status (N1-N2 vs N0)	5.456 (3.329–8.941)	< 0.0001****	2.536 (1.420–4.530)	< 0.0017**
pM status (M1 vs M0)	10.387 (6.121–17.627)	< 0.0001****	4.244 (2.385–7.552)	< 0.0001****
Histological grade (G3 vs G1-G2)	0.773 (0.460–1.297)	0.329		
Recurrence (YES vs NO)	4.149 (2.043–8.425)	< 0.0001****	2.363 (1.155–4.837)	0.019*
GOLT1B expression (High vs Low)	4.092 (1.953–8.573)	< 0.001***	2.194 (1.002–4.802)	0.049*

Univariate and multivariate Cox proportional hazards regression were used to calculate Hazard ratio (HR), 95% confidence intervals (95% CI) and p values in SPSS 24.0. Low GOLT1B is defined as IHC score < 5 and High GOLT1B is defined as IHC score ≥ 5. *p < 0.05, **p < 0.01, ***p < 0.001, ****p < 0.0001

### GOLT1B promotes CRC metastasis in vitro and in vivo

**Fig. 2:**
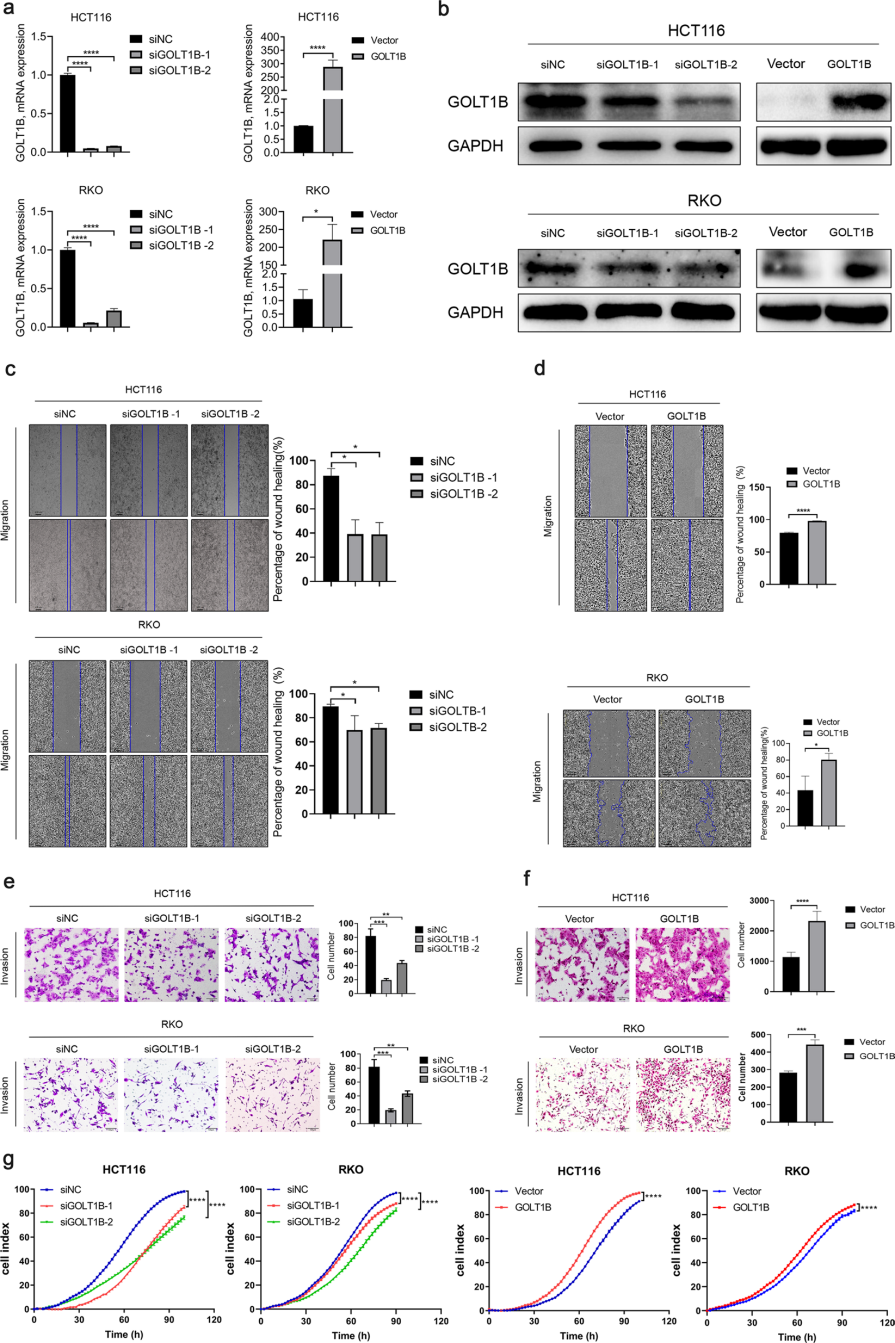
GOLT1B promotes CRC cell proliferation, invasion and migration. **a**, **b** GOLT1B mRNA and protein level were determined by qPCR and western blot in HCT116 and RKO cells. **c**, **d** Cell migration were determined by wound healing experiment after siRNA transfection or overexpression of GOLT1B, respectively. **e**, **f** Cell invasion were detected by transwell assays with matrigel after siRNA transfection or overexpression of GOLT1B, respectively. **g** Effect of GOLT1B on proliferation of colorectal cancer cells in vitro. All data are shown as mean ± S.E.M. *P < 0.05, **P < 0.01, ***P < 0.001, ****P < 0.0001

To investigate the function of GOLT1B in CRC, we first analyzed the relative expression levels of GOLT1B in 11 CRC cell lines (Additional file 3: Fig. S1c) and selected HCT116 and RKO to establish GOLT1B knock-down and overexpression cell lines (Fig. 2a, b). GOLT1B downregulation significantly inhibited CRC cell proliferation, migration and invasion, whereas, GOLT1B overexpression significantly promoted CRC cell growth, migration and invasion (Fig. 2c–g).

**Fig. 3:**
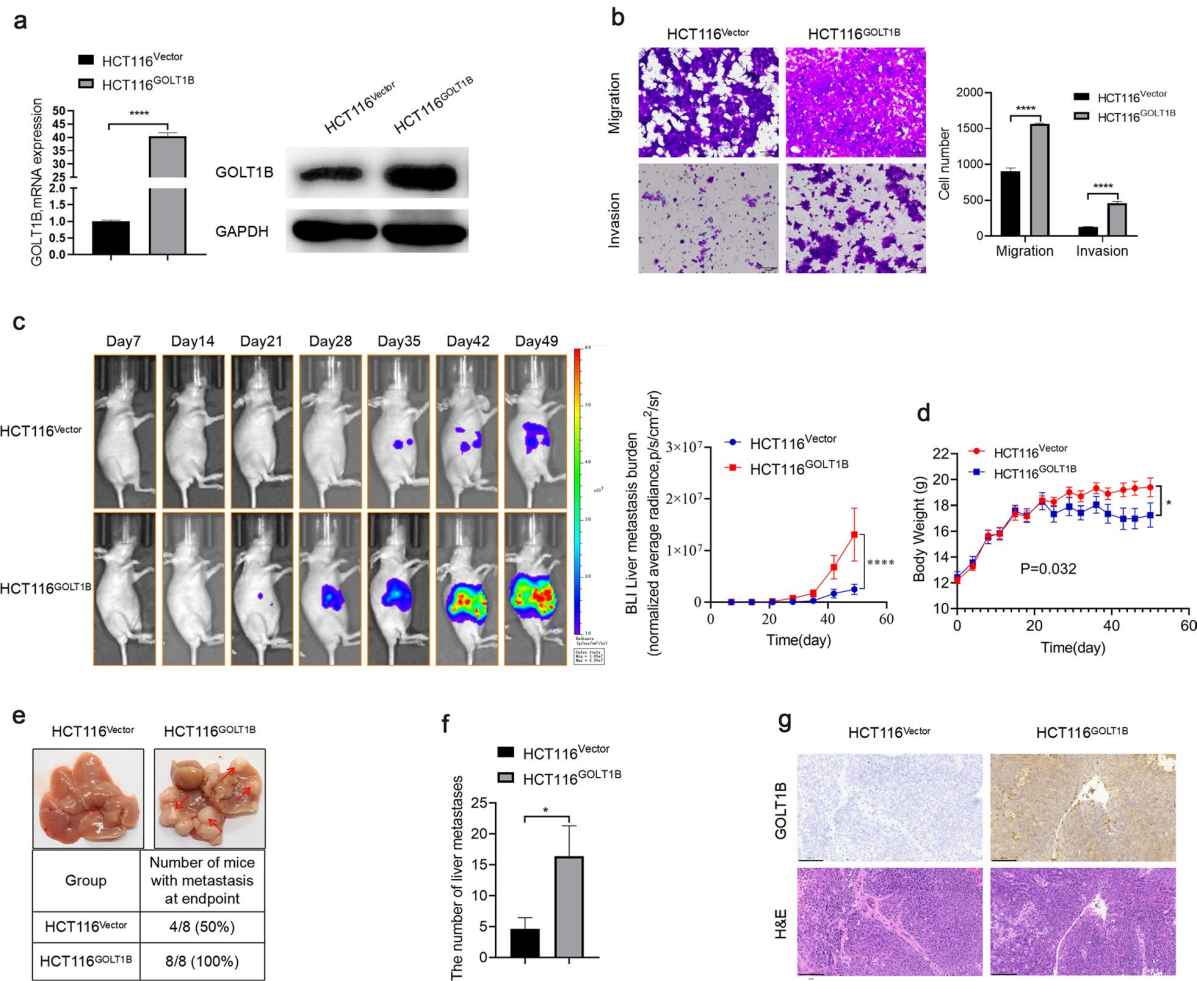
GOLT1B promotes metastasis of colorectal cancer in vivo. **a** GOLT1B stable overexpression was verified by qPCR and western blot. **b** The migration and invasion ability of HCT116 cells stablely transfected with control vector or GOLT1B were determined by transwell assays in vitro. **c** The average bioluminescence imaging signals in mice bearing liver metastatic burden was determined by IVIS imaging (HCT116^Vector^ *vs.* HCT116^GOLT1B^, n = 8). **d** Body weight curves of mice in two groups. **e** Representative images of metastatic foci (pointed out with red arrow) in mice liver. **f** The number of liver metastatic foci in control vector and GOLT1B group. **g** IHC staining of GOLT1B and H&E staining in liver metastatic loci

We next investigated the pro-metastasis function of GOLT1B in vivo. GOLT1B stable overexpression HCT116 cell lines (HCT116^GOLT1B^) and control cell line (HCT116^Vector^) were established. The cell migration and invasion ability were confirmed before in vivo inoculation (Fig. 3a, b). The tumor spread and metastasis was monitored by in vivo imaging system. As shown in Fig. 3c, GOLT1B overexpression group exhibited significantly stronger fluorescence signal than the control group. The average body weight of mice in GOLT1B overexpression group decreased significantly (Fig. 3d). Moreover, HCT116^GOLT1B^ cells showed higher liver metastatic rate and more metastatic foci in liver than HCT116^Vector^ cells (Fig. 3e and f). IHC staining of mouse liver confirmed the overexpression of GOLT1B in liver metastatic foci in HCT116^GOLT1B^ group (Fig. 3g). The above results indicated that high expression of GOLT1B could promote the metastasis of CRC in vivo.

### GOLT1B activates Wnt/β-catenin pathway and induces EMT

**Fig. 4:**
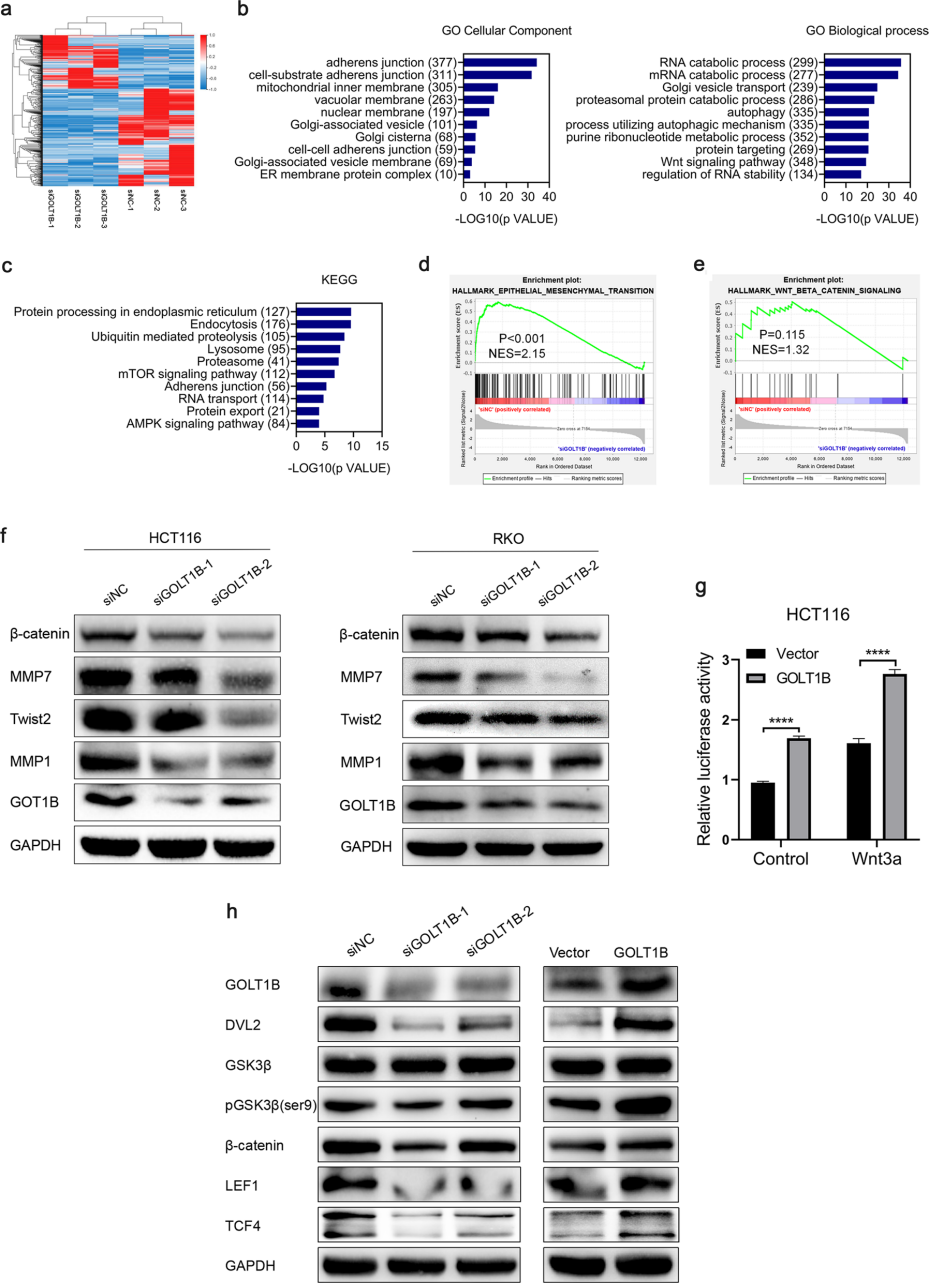
GOLT1B induces EMT via regulating Wnt/β-catenin pathway. **a** Heatmap shows the significantly altered 531 genes (P < 0.05, fold change ≥ 1.5) after knocking down of GOLT1B in HCT116 cells (supplementing the detailed gene in Table S2). **b**, **c** KEGG and GO analyses of differential expression genes on Dr. Tom platform (BGI-tech, China). **d**, **e** GSEA enrichment analysis of differentially expressed genes. **f** EMT-related gene expression was determined by western blot after downregulation of GOLT1B. **g** The dual luciferase report experiment showed that overexpression of GOLT1B in HCT116 can activate the Wnt signaling pathway. **h** Activation of Wnt signaling pathway were analyzed by Western blot analysis

To investigate the intracellular signal pathway related to GOLT1B overexpression in CRC, we performed RNA-seq in control and knocked down GOLT1B HCT116 cells (Fig. 4a, Additional file 4: Table S3). GO analysis confirmed that GOLT1B was closely related to the adhesion function of cells, the vesicle transport of the Golgi complex, protein targeting and Wnt signaling pathway (Fig. 4b) [29]. KEGG analysis indicated that the potential function of GOLT1B was mainly associated with cell adhesion function, protein transport and RNA transport (Fig. 4c) [30]. Gene Set Enrichment Analysis (GSEA) showed that Wnt/β-catenin and EMT-related gene sets were enriched in GOLT1B high expression phenotype. (Fig. 4d, e). The expression of EMT-related proteins decreased after CRC cells knocked down GOLT1B (Fig. 4f) [31, 32]. To determine if GOLT1B overexpression is associated with Wnt/β-catenin signaling activation, we firstly performed a TCF/LEF reporter luciferase assay using HCT116 transfected with GOLT1B together with TCF/LEF-Luc. GOLT1B overexpression significantly enhanced the transcriptional activity of the luciferase reporter, which was more obvious when activated by Wnt3a, suggesting activation of the canonical Wnt pathway (Fig. 4g). Western blot analysis showed that GOLT1B increased the level of β-catenin, DVL2, pGSK3β, LEF1 and TCF4 (Fig. 4h). Furthermore, the results of GEPIA2 analysis showed a significant positive correlation between the expressions of Wnt pathway related proteins β-catenin, DVL2, TCF4, MMP1, MMP7, and CD44 and GOLT1B [33], which were consistent with the results of western blot described above (Additional file 3: Fig. S1d). Therefore, the results suggest that GOLT1B may promote CRC metastasis by activating the Wnt/β-catenin signaling pathway.

### GOLT1B interacts with DVL2 to facilitate its cell membrane localization

**Fig. 5:**
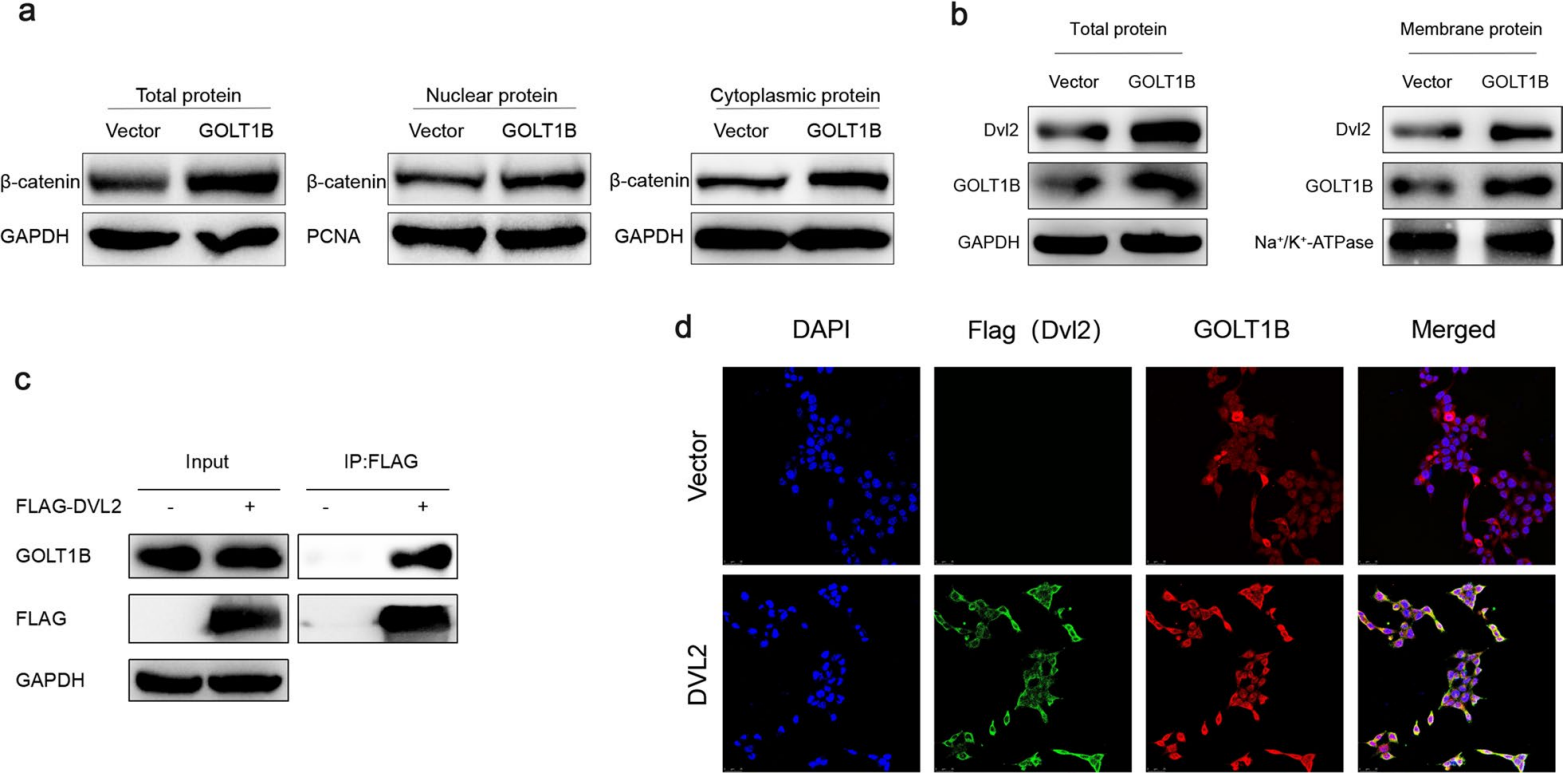
GOLT1B interacts with DVL2 to promote it transport and localization to the cell membrane. **a** The nucleus and cytoplasm separation experiment showed that GOLT1B overexpression increased the level of nuclear β-catenin. **b** The cell membrane and cytoplasm separation experiment showed that GOLT1B could induce DVL2 upregualtion on the cell membrane. **c** CO-IP experiment confirmed the interaction between GOLT1B and DVL2. **d** Immunofluorescence experiment showed that GOLT1B and DVL2 were co-localized

During the activation of Wnt/β-catenin signaling pathway, DVL2 is recruited onto the cell membrane binding the C-terminal of Frizzled, which then destroy the APC-GSK3β complex and induce the nuclear accumulation of β-Catenin [34–36]. As GOLT1B is a Golgi complex vesicle transporter, we speculated that whether GOLT1B is involved in the transportation of DVL2 and β-catenin, the two critical molecules on Wnt pathway. The separation of nucleus and cytoplasm experiment confirmed that the overexpression of GOLT1B did increase the level of β-catenin in the nucleus (Fig. 5a). Also, the membrane expression of DVL2 was increased upon GOLT1B overexpression (Fig. 5b). However, CO-IP experiment only showed obvious interaction between GOLT1B and DVL2 (Fig. 5c). Furthermore, immunofluorescence experiments also confirmed the spatial distribution co-localization of GOLT1B and DVL2 (Fig. 5d). According to the above results, we speculated that GOLT1B may interact with DVL2 and facilitate its transportation to the cell membrane, thereby activating the downstream Wnt/β-catenin signaling.

### GOLT1B regulates the expression level of PD-L2 to induce tumor immune escape

**Fig. 6:**
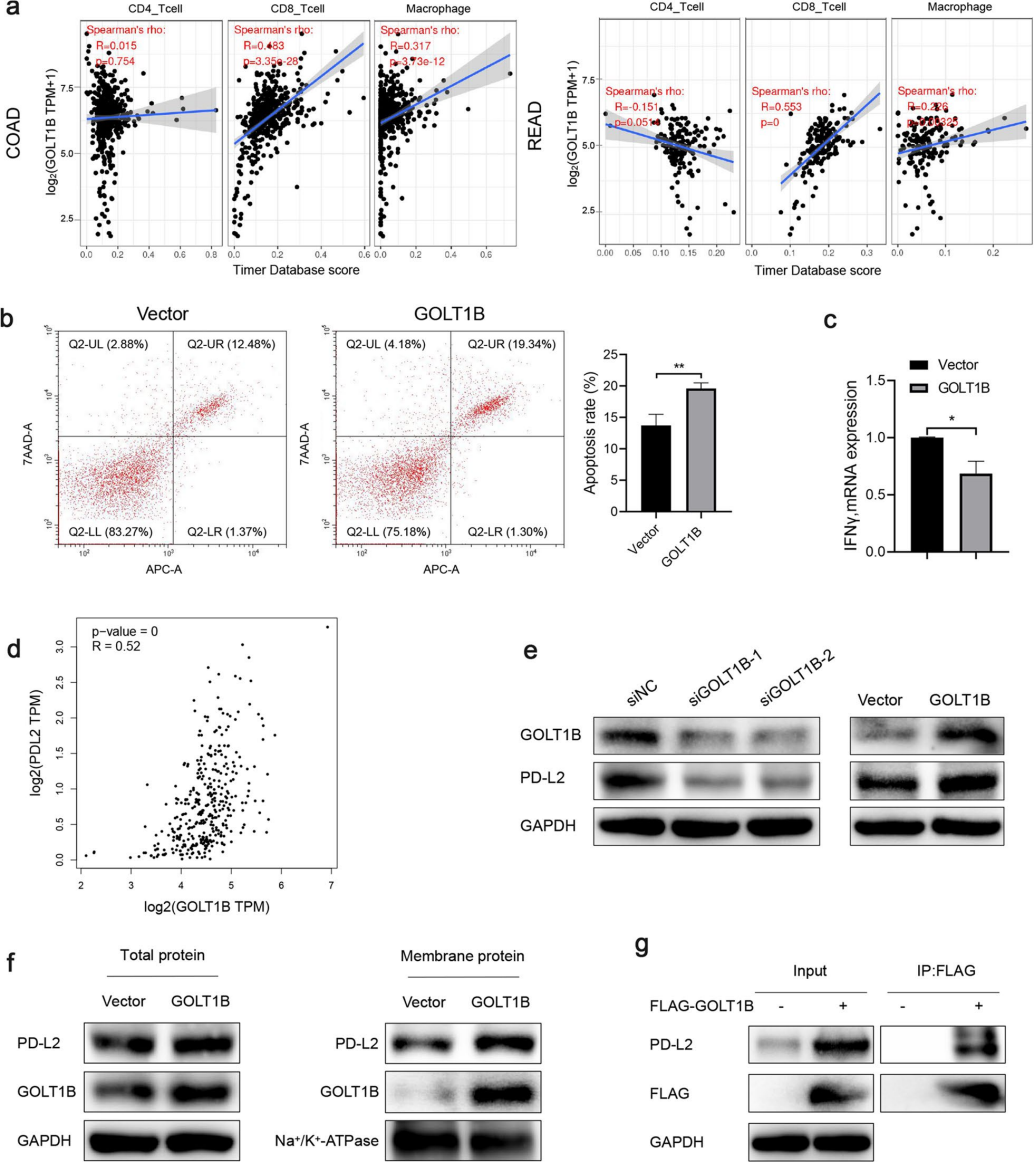
GOLT1B regulates the expression and membrane translation of PD-L2. **a** The correlation between GOLT1B and immuno-infiltrating cells in CRC from TIMER database. **b** Co-culture of HCT116 and Jurkat cell showed that GOLT1B overexpression in cancer cells could induce Jurkat cells apoptosis and **c** inhibit the IFN-γ level in Jurkat cell. **d** The correlation between PD-L2 and GOLT1B in CRC from GEPIA2 database. **e** GOLT1B knockdown or overexpression could decrease and increase PD-L2 level in CRC cells, respectively. **f** The cell membrane and cytoplasm separation experiment showed that GOLT1B could upregulate the PD-L2 level on the cell membrane. **g** The CO-IP experiment confirmed the interaction between GOLT1B and PD-L2

Based on the analysis of online database TIMER (Tumor IMmune Estimation Resource) [37], we found that GOLT1B was significantly associated with the infiltration of CD4+ T cells, CD8+ T cells and macrophages. Therefore, we speculated that GOLT1B might be involved in the tumor immune response (Fig. 6a). Next, we overexpressed GOLT1B in HCT116 and directly cocultured with activated Jurkat cell, followed by flow cytometry analysis of Jurkat cell apoptosis. The results showed that the coculture of HCT116 overexpressing GOLT1B and Jurkat cell significantly promoted the apoptosis of Jurkat cell, and the expression level of IFNγ in Jurkat cell was also significantly reduced (Fig. 6b, c). Then through online analysis of the GEPIA2 database, a significant correlation was found between GOLT1B and PD-L2 (Fig. 6d). Western blot analysis showed that the expression of PD-L2 was significantly decreased after the knock-down of GOLT1B, while the expression of PD-L2 was significantly increased after the over-expression of GOLT1B in HCT116 (Fig. 6e). Cell membrane and cytoplasm separation experiments showed that GOLT1B overexpression could result in an increase of cell membrane PD-L2 level (Fig. 6f). Moreover, CO-IP experiments confirmed the existence of interaction between GOLT1B and PD-L2 (Fig. 6g). Based on these results, we believe that GOLT1B can promote immune escape by increasing the expression level of PD-L2 in cancer cells and promoting apoptosis of T lymphocytes in the tumor microenvironment, which further promotes the metastasis of CRC.

Taken together, GOLT1B may promote CRC metastasis not only by activating the downstream Wnt/β-catenin signaling, but also by facilitating the membrane localization of PD-L2 to induce T lymphocytes apoptosis and thus reshape the tumor microenvironment.

### GOLT1B siRNA suppresses tumor progression in PDX model

**Fig. 7:**
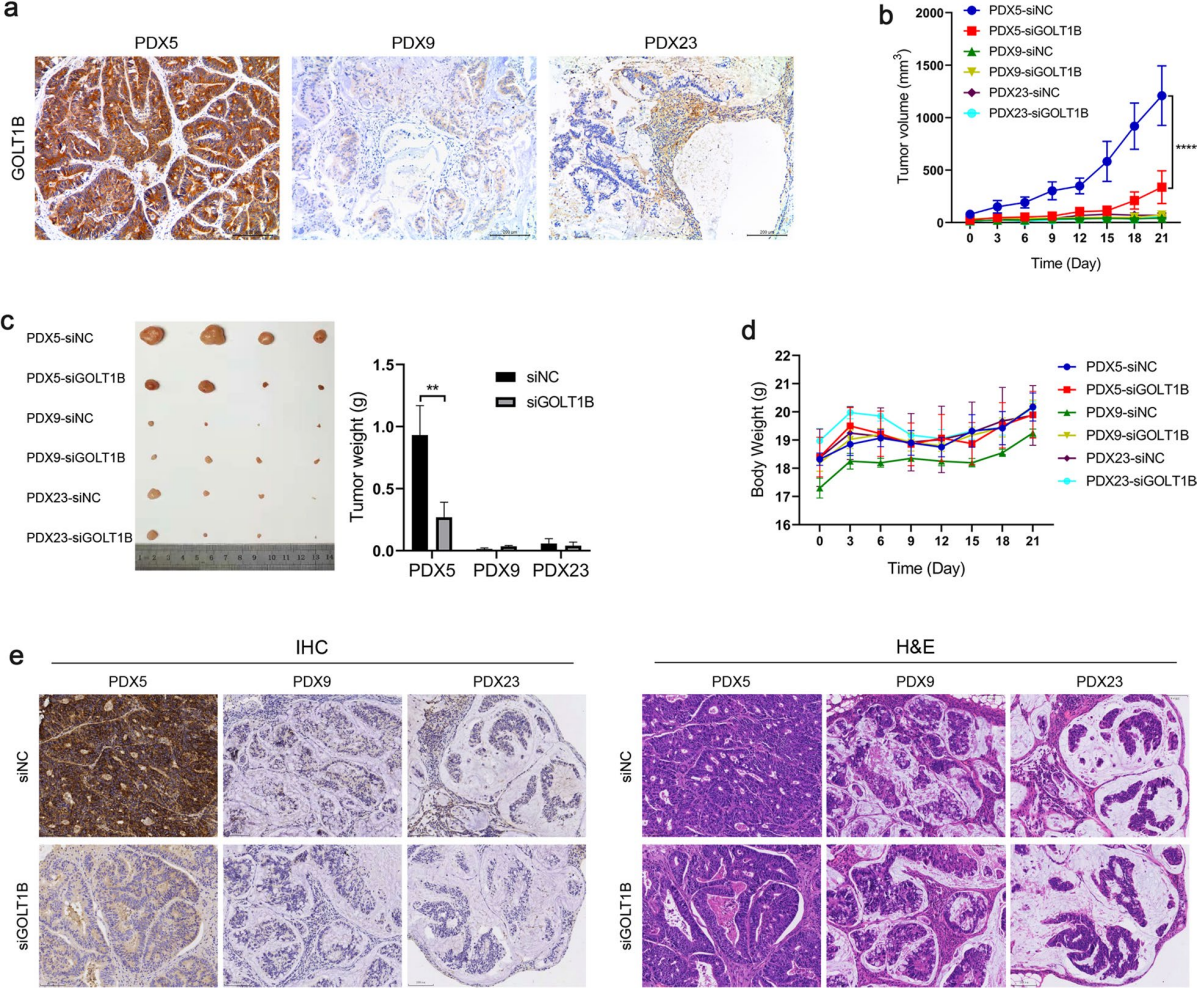
GOLT1B siRNA suppresses tumor progression in PDX model. **a** GOLT1B level in 3 cases of PDX was determined by IHC. **b** Tumor growth curve after siRNA treatment (n = 4 for each PDX). **c** Images of tumors and tumor weight in different PDX after treatment with siRNA or siNC. **d** Body weight curves of each group. **e** IHC and HE staining of tumor in the PDX model

In order to verify whether targeting GOLT1B can suppress cancer progression, CRC PDX model were established, and then siGOLT1B were intraperitoneally injected 4 weeks after tumor inoculation. IHC staining showed that GOLT1B level was significantly higher in PDX5 than that in PDX9 and PDX 23 (Fig. 7a). Accordingly, the tumor growth rate were significantly faster in PDX5. Notably, PDX5 shrank quickly after siGOLT1B treatment, while the tumor volume and weight in PDX9 and PDX23 did not change significantly (Fig. 7b, c). The mice body weight were not significant different (Fig. 7d). The IHC and HE staining showed that after siGOLT1B treatment, the expression level of GOLT1B in the tumor was significantly reduced (Fig. 7e). These results showed that targeting GOLT1B in GOLT1B highly expressed CRC could block tumor progression in PDX model.

## Discussion

Metastasis is the major cause of death in CRC. The effect of intracellular vesicle transporters in cancer development, especially those produced by the Golgi complex, the site of protein maturation, was rarely studied [38–40]. In the present study, we have found GOLT1B is significantly associated with poor prognosis in CRC. In terms of mechanism, GOLT1B assists the transportation of DVL2 to the cell membrane through its interaction with DVL2, thereby inhibiting the activity of GSK3β and β-catenin degradation [41, 42]. Nuclear accumulated β-catenin can bind to the LEF/TCF transcription factor family, which then initiate the transcription of downstream target genes and induces EMT [43–45]. In addition, the RNA-seq analysis showed that GOLT1B was also related to AMPK and mTOR pathways enrichment, which are correlated to cell metabolism and cell survival. Accordingly, our in vitro and in vivo experiments showed that GOLT1B is correlated with cell proliferation, targeting GOLT1B could suppress the tumor growth in PDX model. The underlying mechanism of GOLT1B in regulating CRC progression needs further investigation.

PD-L1 and PD-L2 are the two ligands of PD-1. Studies have shown that the activation of GSK3β-β-TrCP axis can induce the ubiquitination and proteasome degradation of non-glycosylated PD-L1 in mouse breast cancer model [34, 46]. GSK3β inhibitors can increase the expression of PD-L1 [47, 48]. Moreover, inhibiting the Wnt signaling pathway, the expression level of PD-L1 could be decreased in triple-negative breast cancer [49]. However, little is known about the association between Wnt signaling and PD-L2. Our study showed that high expression of GOLT1B induced the increase of PD-L2 expression in CRC cells, which could induce the apoptosis of tumor-infiltrating T lymphocytes and inhibit the level of IFNγ [50]. We speculated that GOLT1B might increase PD-L2 level by inhibiting GSK3β phosphorylation and downstream degradation of PD-L2. Additionally, GOLT1B could also interact with PD-L2 to assist its transportation and localization to the cell membrane to exhibit its function.

**Fig. 8:**
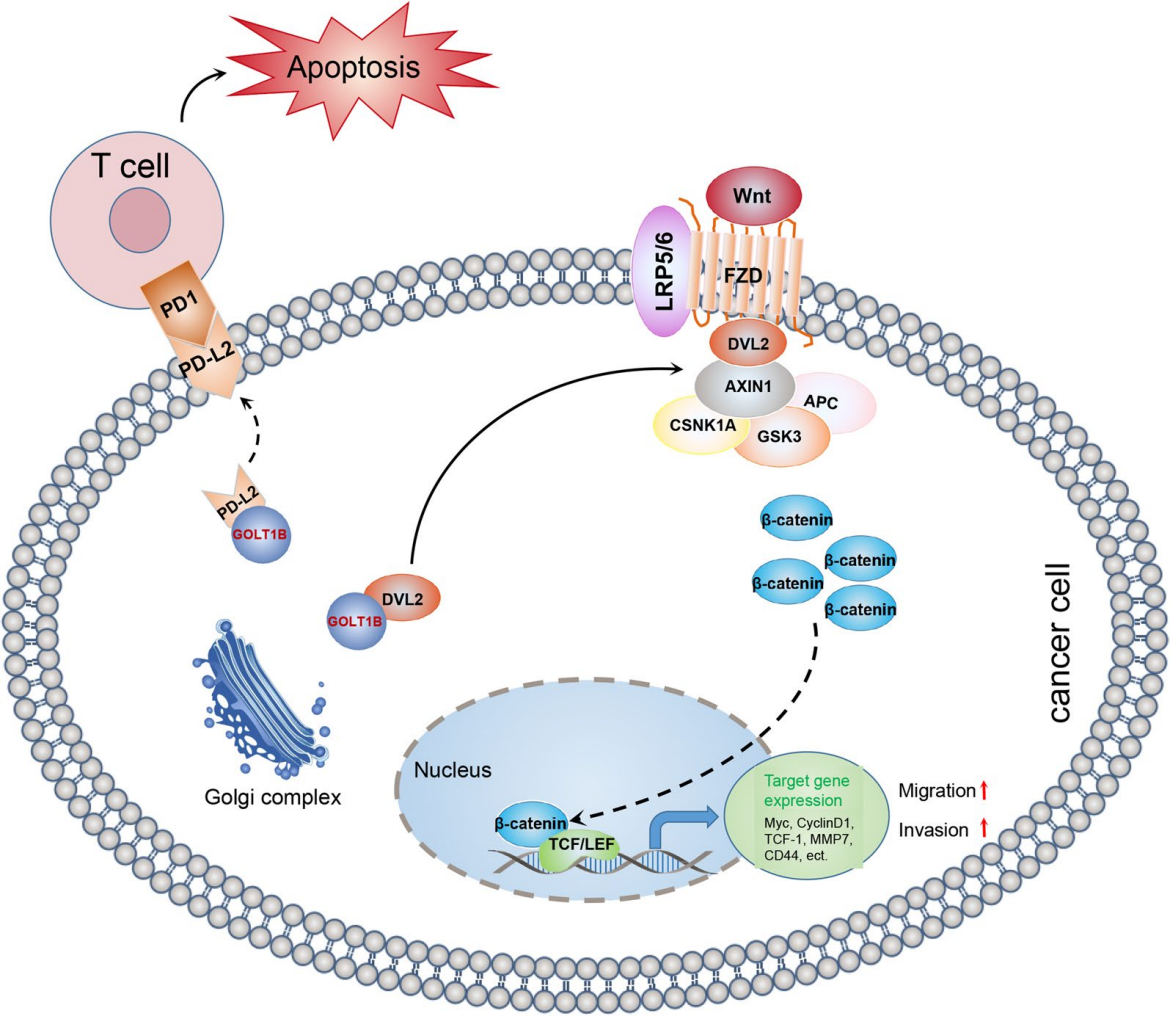
A schematic model of GOLT1B functions in tumor metastasis

In conclusion, our study provides evidence that GOLT1B is an independent prognostic marker in CRC. GOLT1B promotes CRC metastasis via interaction with DVL2 and activating the downstream Wnt/β-catenin signaling. Otherwise, GOLT1B can induce T-cell apoptosis by regulating PD-L2 membrane translocation (Fig. 8). Targeting GOLT1B inhibited tumor progression in PDX model. Our study provides new evidence that targeting GOLT1B may be an effective strategy in CRC treatment.

## Supplementary Information

**Additional file 1: Table S1.** The list of primers and siRNA sequence.

**Additional file 2: Table S2.** The RNA-seq sequencing data of GOLT1B siRNA samples.

**Additional file 3: Figure S1**. GOLT1B is highly expressed in colorectal cancer and regulates cancer cell metastasis through WNT signaling pathway. a. GOLT1B is generally highly expressed in various tumors. b. CPTAC (clinical proteomic tumor analysis consortium) database analysis find that GOLT1B is highly expressed in CRC. c. qPCR analysis of GOLT1B expression in 11 common colorectal cancer cell lines. d. GEPIA2 database analyzes the correlation between GOLT1B and wnt signaling pathway related proteins.

**Additional file 4: Table S3.** The pathological information of CRC patients used in PDX model.

## References

[1] Sung H, Ferlay J, Siegel RL, Laversanne M, Soerjomataram I, Jemal A (2021). Global cancer statistics 2020: GLOBOCAN estimates of incidence and mortality worldwide for 36 cancers in 185 countries. Cancer J Clin..

[2] Siegel RL, Miller KD, Jemal A (2020). Cancer statistics, 2020. CA Cancer J Clin.

[3] MacDonald BT, He X (2012). Frizzled and LRP5/6 receptors for Wnt/beta-catenin signaling. Cold Spring Harb Perspect Biol..

[4] Ohata S, Nakatani J, Herranz-Perez V, Cheng J, Belinson H, Inubushi T (2014). Loss of Dishevelleds disrupts planar polarity in ependymal motile cilia and results in hydrocephalus. Neuron.

[5] Pardoll DM (2012). The blockade of immune checkpoints in cancer immunotherapy. Nat Rev Cancer.

[6] Tanegashima T, Togashi Y, Azuma K, Kawahara A, Ideguchi K, Sugiyama D (2019). Immune suppression by PD-L2 against spontaneous and treatment-related antitumor immunity. Clin Cancer Res.

[7] Yi M, Niu M, Xu L, Luo S, Wu K (2021). Regulation of PD-L1 expression in the tumor microenvironment. J Hematol Oncol.

[8] Xing X, Guo J, Ding G, Li B, Dong B, Feng Q (2018). Analysis of PD1, PDL1, PDL2 expression and T cells infiltration in 1014 gastric cancer patients. Oncoimmunology..

[9] Keir ME, Butte MJ, Freeman GJ, Sharpe AH (2008). PD-1 and its ligands in tolerance and immunity. Annu Rev Immunol.

[10] Zengin M, Zergeroglu S, Okcu O, Benek S (2021). PD-1 and PD-L2 expression predict relapse risk and poor survival in patients with stage III colorectal cancer. Cell Oncol..

[11] Fan F, Chen K, Lu X, Li A, Liu C, Wu B (2020). Dual targeting of PD-L1 and PD-L2 by PCED1B-AS1 via sponging hsa-miR-194–5p induces immunosuppression in hepatocellular carcinoma. Hepatol Int..

[12] Zizzari IG, Di Filippo A, Scirocchi F, Di Pietro FR, Rahimi H, Ugolini A, et al. Soluble Immune Checkpoints, Gut Metabolites and Performance Status as Parameters of Response to Nivolumab Treatment in NSCLC Patients. J Pers Med. 2020;10(4). 10.3390/jpm10040208 PMC7712566 33158018

[13] Al Hadidi SA, Lee HJ (2020). Pembrolizumab for the treatment of Hodgkin Lymphoma. Expert Opin Biol Ther.

[14] Hiraoka N, Ino Y, Hori S, Yamazaki-Itoh R, Naito C, Shimasaki M (2020). Expression of classical human leukocyte antigen class I antigens, HLA-E and HLA-G, is adversely prognostic in pancreatic cancer patients. Cancer Sci.

[15] Li H, Yang LL, Xiao Y, Deng WW, Chen L, Wu L (2018). Overexpression of golgi phosphoprotein 2 is associated with poor prognosis in oral squamous cell carcinoma. Am J Clin Pathol.

[16] Riener MO, Stenner F, Liewen H, Soll C, Breitenstein S, Pestalozzi BC (2009). Golgi phosphoprotein 2 (GOLPH2) expression in liver tumors and its value as a serum marker in hepatocellular carcinomas. Hepatology.

[17] Byrne AM, Bekiaris S, Duggan G, Prichard D, Kirca M, Finn S (2015). Golgi phosphoprotein 2 (GOLPH2) is a novel bile acid-responsive modulator of oesophageal cell migration and invasion. Br J Cancer.

[18] Wu F, Gao P, Wu W, Wang Z, Yang J, Di J (2018). STK25-induced inhibition of aerobic glycolysis via GOLPH3-mTOR pathway suppresses cell proliferation in colorectal cancer. J Exp Clin Cancer Res.

[19] Wang Z, Jiang B, Chen L, Di J, Cui M, Liu M (2014). GOLPH3 predicts survival of colorectal cancer patients treated with 5-fluorouracil-based adjuvant chemotherapy. J Transl Med.

[20] Abraham RT (2009). GOLPH3 links the Golgi network to mTOR signaling and human cancer. Pigment Cell Melanoma Res.

[21] Ye QH, Zhu WW, Zhang JB, Qin Y, Lu M, Lin GL (2016). GOLM1 modulates EGFR/RTK cell-surface recycling to drive hepatocellular carcinoma metastasis. Cancer Cell.

[22] Liu Y, Zhou S, Shi J, Zhang X, Shentu L, Chen Z (2019). c-Myc transactivates GP73 and promotes metastasis of hepatocellular carcinoma cells through GP73-mediated MMP-7 trafficking in a mildly hypoxic microenvironment. Oncogenesis.

[23] Ikeda K, Horie-Inoue K, Ueno T, Suzuki T, Sato W, Shigekawa T (2015). miR-378a-3p modulates tamoxifen sensitivity in breast cancer MCF-7 cells through targeting GOLT1A. Sci Rep.

[24] Zhang L, Hu R, Cheng Y, Wu X, Xi S, Sun Y (2017). Lidocaine inhibits the proliferation of lung cancer by regulating the expression of GOLT1A. Cell Proli..

[25] Huang J, Liang X, Xuan Y, Geng C, Li Y, Lu H (2017). A reference human genome dataset of the BGISEQ-500 sequencer. Gigascience.

[26] Chandrashekar DS, Bashel B, Balasubramanya SAH, Creighton CJ, Ponce-Rodriguez I, Chakravarthi B (2017). UALCAN: a portal for facilitating tumor subgroup gene expression and survival analyses. Neoplasia.

[27] Rhodes DR, Yu J, Shanker K, Deshpande N, Varambally R, Ghosh D (2004). ONCOMINE: a cancer microarray database and integrated data-mining platform. Neoplasia.

[28] Edwards NJ, Oberti M, Thangudu RR, Cai S, McGarvey PB, Jacob S (2015). The CPTAC data portal: a resource for cancer proteomics research. J Proteome Res.

[29] Ashburner M, Ball CA, Blake JA, Botstein D, Butler H, Cherry JM (2000). Gene ontology: tool for the unification of biology. The Gene Ontology Consortium Nat Genet.

[30] Ogata H, Goto S, Sato K, Fujibuchi W, Bono H, Kanehisa M (1999). KEGG: Kyoto encyclopedia of genes and genomes. Nucleic Acids Res.

[31] Dongre A, Weinberg RA (2019). New insights into the mechanisms of epithelial-mesenchymal transition and implications for cancer. Nat Rev Mol Cell Biol.

[32] Lamouille S, Xu J, Derynck R (2014). Molecular mechanisms of epithelial-mesenchymal transition. Nat Rev Mol Cell Biol.

[33] Tang Z, Kang B, Li C, Chen T, Zhang Z (2019). GEPIA2: an enhanced web server for large-scale expression profiling and interactive analysis. Nucleic Acids Res.

[34] Galluzzi L, Spranger S, Fuchs E, Lopez-Soto A (2019). WNT signaling in cancer immunosurveillance. Trends Cell Biol.

[35] Nusse R, Clevers H (2017). Wnt/beta-Catenin signaling, disease, and emerging therapeutic modalities. Cell.

[36] Clevers H, Nusse R (2012). Wnt/beta-catenin signaling and disease. Cell.

[37] Li T, Fan J, Wang B, Traugh N, Chen Q, Liu JS (2017). TIMER: a web server for comprehensive analysis of tumor-infiltrating immune cells. Cancer Res.

[38] Zhao S, Mi Y, Guan B, Zheng B, Wei P, Gu Y (2020). Tumor-derived exosomal miR-934 induces macrophage M2 polarization to promote liver metastasis of colorectal cancer. J Hematol Oncol.

[39] Guo S, Chen J, Chen F, Zeng Q, Liu WL, Zhang G (2020). Exosomes derived from Fusobacterium nucleatum-infected colorectal cancer cells facilitate tumour metastasis by selectively carrying miR-1246/92b-3p/27a-3p and CXCL16. Gut..

[40] Shang A, Gu C, Wang W, Wang X, Sun J, Zeng B (2020). Exosomal circPACRGL promotes progression of colorectal cancer via the miR-142-3p/miR-506-3p- TGF-beta1 axis. Mol Cancer.

[41] Chen W, ten Berge D, Brown J, Ahn S, Hu LA, Miller WE (2003). Dishevelled 2 recruits beta-arrestin 2 to mediate Wnt5A-stimulated endocytosis of Frizzled 4. Science.

[42] Kozielewicz P, Turku A, Bowin CF, Petersen J, Valnohova J, Canizal MCA (2020). Structural insight into small molecule action on Frizzleds. Nat Commun.

[43] Zhang Y, Wang X (2020). Targeting the Wnt/beta-catenin signaling pathway in cancer. J Hematol Oncol.

[44] Santos R, Linker SB, Stern S, Mendes APD, Shokhirev MN, Erikson G (2021). Deficient LEF1 expression is associated with lithium resistance and hyperexcitability in neurons derived from bipolar disorder patients. Mol Psychiatry..

[45] Zhang M, Lai Y, Krupalnik V, Guo P, Guo X, Zhou J (2020). beta-Catenin safeguards the ground state of mousepluripotency by strengthening the robustness of the transcriptional apparatus. Sci Adv..

[46] Li CW, Lim SO, Xia W, Lee HH, Chan LC, Kuo CW (2016). Glycosylation and stabilization of programmed death ligand-1 suppresses T-cell activity. Nat Commun.

[47] Capietto AH, Kim S, Sanford DE, Linehan DC, Hikida M, Kumosaki T (2013). Down-regulation of PLCgamma2-beta-catenin pathway promotes activation and expansion of myeloid-derived suppressor cells in cancer. J Exp Med.

[48] Li H, Li CW, Li X, Ding Q, Guo L, Liu S (2019). MET inhibitors promote liver tumor evasion of the immune response by stabilizing PDL1. Gastroenterology..

[49] Castagnoli L, Cancila V, Cordoba-Romero SL, Faraci S, Talarico G, Belmonte B (2019). WNT signaling modulates PD-L1 expression in the stem cell compartment of triple-negative breast cancer. Oncogene.

[50] Riley JL (2009). PD-1 signaling in primary T cells. Immunol Rev.

