# Supplementary material for: Vesicle transporter GOLT1B mediates the cell membrane localization of DVL2 and PD-L2 and promotes colorectal cancer metastasis
Source: Cancer Cell Int. 2021 May 31;21:287. doi: 10.1186/s12935-021-01991-z (PMC8166103; doi:10.1186/s12935-021-01991-z)
Supplement: Supplementary file 3 — Additional file 3: Figure S1. GOLT1B is highly expressed in colorectal cancer and regulates cancer cell metastasis through WNT signaling pathway. a. GOLT1B is generally highly expressed in various tumors. b. CPTAC (clinical proteomic tumor analysis consortium) database analysis find that GOLT1B is highly expressed in CRC. c. qPCR analysis of GOLT1B expression in 11 common colorectal cancer cell lines. d. GEPIA2 database analyzes the correlation between GOLT1B and wnt signaling pathway related proteins. [file 12935_2021_1991_MOESM3_ESM.docx]

**Supplementary Materials**


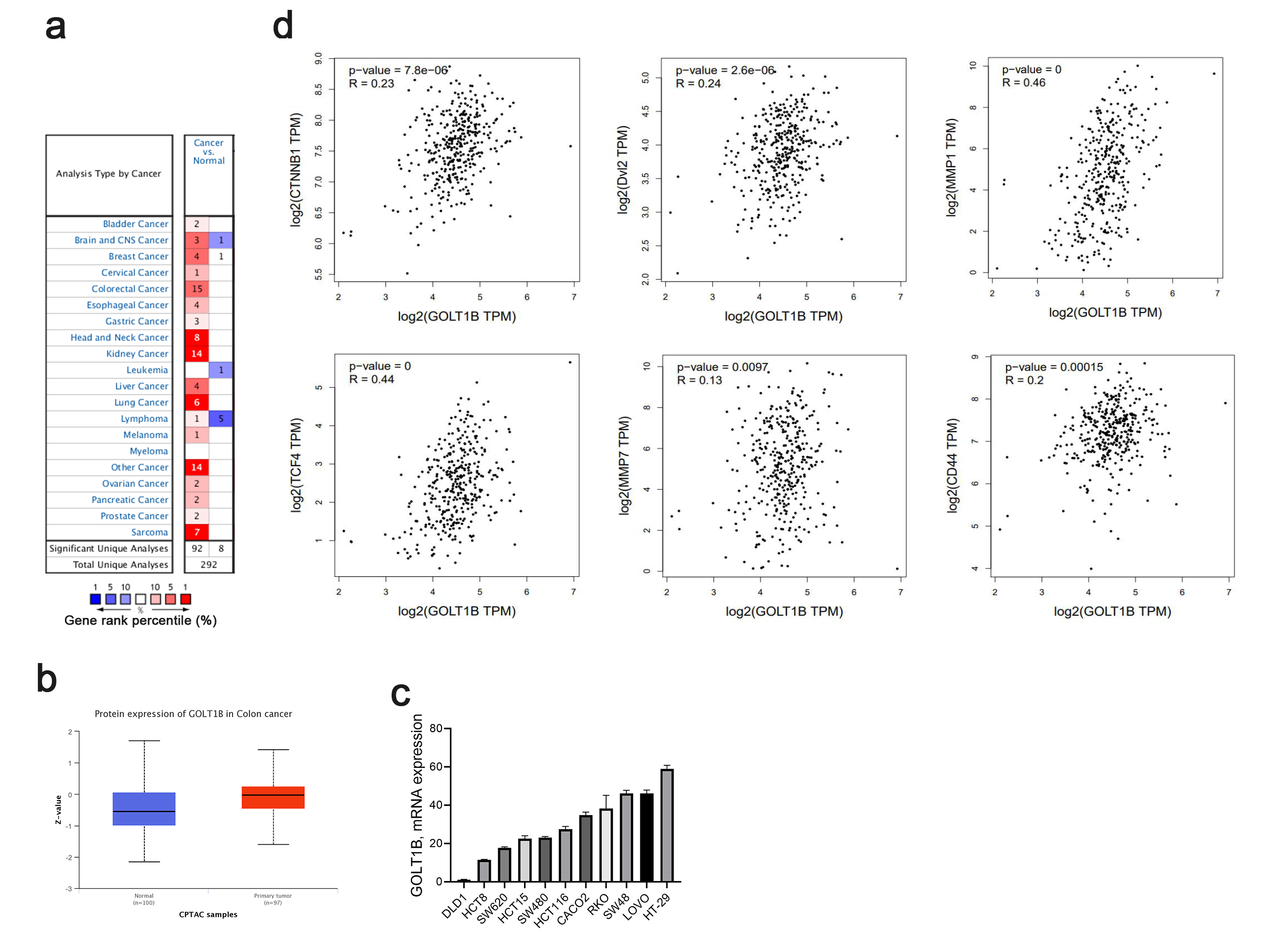


**Supplementary** **Figure.S1** **GOLT1B is highly expressed in colorectal cancer and regulates cancer cell metastasis through WNT signaling pathway. a.** GOLT1B is generally highly expressed in various tumors. **b.** CPTAC (clinical proteomic tumor analysis consortium) database analysis find that GOLT1B is highly expressed in CRC. **c.** QPCR analysis of GOLT1B expression in 11 common colorectal cancer cell lines. **d.** GEPIA2 database analyzes the correlation between GOLT1B and wnt signaling pathway related proteins.
